# Physiological and Behavioral Response Differences Between Video-Mediated and In-Person Interaction

**DOI:** 10.3390/s26010034

**Published:** 2025-12-20

**Authors:** Christoph Tremmel, Nathan T. M. Huneke, Daniel Hobson, Christopher Tacca, m.c. schraefel

**Affiliations:** 1 Electronics and Computer Science, University of Southampton, Southampton SO17 1BJ, UK; 2 Clinical and Experimental Sciences, Faculty of Medicine, University of Southampton, Southampton SO16 6YD, UK; 3 Institute for Life Sciences, University of Southampton, Southampton SO17 1BJ, UK

**Keywords:** video-mediated communication, biomedical sensors, human–computer interaction

## Abstract

This study investigates how virtual communication differs from in-person interaction across physiological and behavioral domains, with the goal of informing future interface design. Using a naturalistic setup, we recorded multimodal biosignals, including eye tracking, head and hand movement, heart rate, respiratory rate, and EEG during both in-person and video-based dialogues. Our results show that virtual communication significantly reduces movement and gaze dynamics, particularly in horizontal eye movements and lateral head motion, reflecting both sender- and receiver-side constraints. These physical limitations likely stem from the need to remain within the camera frame and the restricted access to nonverbal cues. Pupil dilation was significantly greater during in-person conversations, consistent with increased arousal during natural communication. Heart rate and EEG trends similarly suggested heightened engagement in face-to-face settings, though interpretation of EEG was limited by movement artifacts. Together, the findings highlight how virtual platforms alter embodied interaction, underscoring the need to address both mobility and visual access in future communication technologies to better support co-presence.

## 1. Introduction

The rise of digital communication technologies has resulted in a substantial increase in the use of virtual meetings in both professional and personal settings [1,2,3]. The COVID-19 pandemic significantly accelerated this shift [4,5] by driving the development of more advanced virtual meeting platforms, improving internet reliability and speed [6], reducing travel-related costs and, in some cases, increasing productivity when in-person travel was replaced by online meetings [7,8,9]. It also broadened opportunities for remote collaboration with international teams [3]. In addition, many employees have expressed a preference for remote work in part due to the opportunity for remote work to facilitate better work–life balance (rated more important than job security in 2025 [10]), and in part due to concerns about the environmental impact of carbon emissions arising from in-person travel [11]. In addition to its workplace benefits, virtual communication promotes accessibility and inclusion, especially for individuals with disabilities or neurodivergent conditions [12]. It also offers greater flexibility and convenience compared to in-person meetings [13]. Moreover, certain features are unique to virtual platforms, such as the ability to record conversations for later review.

Despite the established benefits, virtual communication presents challenges that have been shown to limit its effectiveness compared to in-person interaction. In comparison to in-person meetings, virtual meetings have lower group cohesion and reduced satisfaction with how interactions unfold [14,15]; a weaker sense of social presence and increased feelings of isolation due to reduced contact with colleagues [15,16,17]; and increased emotional fatigue caused by longer working hours and the expectation to be constantly available [18]. Trust between participants can also be harder to establish virtually, as research has repeatedly shown that trust often develops more naturally in face-to-face settings [14,15]. A study comparing videoconferencing, in-person communication, and virtual reality found videoconferencing to be the least engaging of the three communication methods investigated [19]. In addition, some users report increased anxiety from seeing their own face presented during calls [20,21] or struggle with information overload [22] referred to as “Zoom fatigue”, a form of exhaustion specific to video conferencing [23,24]. Technical issues can further contribute to this phenomenon, particularly in non-professional or everyday environments where users may lack specialized support. Common problems include unstable internet connections, poor audio or video quality, and persistent concerns about privacy and data security [25]. Importantly, these effects are not uniformly experienced by users, complicating the design of universally effective interfaces. While average patterns can be identified, individual responses to remote communication vary widely. For instance, employees with negative perceptions of workplace relationships may be more likely to prefer remote interaction [26].

Although the aforementioned differences between virtual and in-person interaction are well documented, the behavioral and physiological mechanisms behind them are not yet well understood. Previous studies have focused on isolated aspects of communication, such as face viewing [27,28], text-based chat [29], or lecture-style video conferencing [30], often using constrained tasks and single signal modalities, which limits their applicability to natural social interaction.

### 1.1. Subject of the Study

To move beyond these limitations, we investigated how both behavioral and physiological signals differ across communication modalities during unscripted, face-to-face-style conversations. We simultaneously recorded both physiological and behavioral signals, enabling a comprehensive analysis of how communication modality influences embodied and neural responses. The recorded signals include eye tracking, head and body movement, heart rate, respiratory rate, and EEG. Unlike prior studies, our design involved unstructured, dyadic conversation to more closely approximate real social encounters. In addition, we interpret physiological and behavioral changes through the lens of both sender and receiver constraints, identifying which limitations of virtual platforms most affect interaction quality—and where design interventions could be most impactful.

### 1.2. Motivation

Our motivating hypothesis is that by better understanding the physiological and behavioral differences between in-person and digitally mediated communication, we can design better digitally mediated interactions informed by those models to enhance the sense of co-presence, the feeling of being physically together, and ultimately improve virtual communication. We anticipate that such models that can be translated into interaction designs will help virtual meetings better support natural dynamics, with potential benefits for user experience, trust, collaboration, and sustainability.

Because interaction is shaped by complex and interdependent behavioral and physiological processes, we argue that modifying a single variable (e.g., heart rate) is unlikely to meaningfully improve co-presence. Instead, we hypothesize that mapping multiple biosignals can reveal patterns shaped by latent interaction constraints, offering more effective targets for future design interventions.

### 1.3. Contribution of This Study

The key contribution of this work is a multimodal analysis of in-person versus virtual communication during unscripted naturalistic interaction from both the sender and the receiver perspectives. To our knowledge, no other study has analyzed in-person versus virtual communication in this way.

### 1.4. Paper Structure

In this paper, we first discuss the state of the art of in-person versus virtual communication analysis. Then, we explain our approach for the analysis, including the experimental task, experimental procedure, data recording, pre-processing, and feature extraction. Following this, we report on the results of the study and discuss its implications towards better understanding virtual communication. Finally, we conclude with a summary of the overall contributions of this study to the field and opportunities for further research.

## 2. State of the Art

When two persons engage in a conversation, they do more than simply exchange words. People coordinate on multiple levels, forming a dynamic, self-organizing system influenced by interpersonal synergy, where meaning arises through complementary and context-sensitive interactions tailored to the situation [31]. During dialogues, participants often synchronize (or mirror) physical behaviors, such as body and head motions [32], eye movements [33], and breathing patterns [34]. This coordination signals affiliation and rapport, and enhances communication effectiveness, learning, and social connection [35]. Comparable forms of interpersonal synchrony have also been observed in physiological signals, such as heart rate (HR) [36] and galvanic skin response (GSR) [37], as well as in neural activity measured through electroencephalography (EEG) [38] or functional near-infrared spectroscopy (fNIRS) [39], using hyperscanning, in which brain activity from multiple individuals is recorded simultaneously.

From this overview, we can see that current, predominantly video-mediated, virtual communication lacks several key components of in-person interaction. Perhaps the most widely explored is that, in face-to-face settings, individuals shift their gaze across the face, hands, and body of their conversation partner [40], using a wide range of non-verbal signals, such as gestures and eye contact, to enrich and guide communication [41,42]. Video calls, in contrast, narrow the visual field of view and disrupt natural eye contact due to camera placement. This narrowing reduces gesture visibility and impairs turn-taking and expression clarity. This has been shown to contribute to lower movement synchrony online [43]. Facial expressiveness is also affected, though this varies across individuals [44]. Physical constraints during virtual meetings can also reduce mobility, which may negatively affect cognitive functioning and communication quality [23,45]. Subjective reports also show that arousal levels are significantly lower during virtual communication [29]. Additionally, a recent study found that making hand gestures visible during online meetings led to more positive participant feedback [46], suggesting that enhancing nonverbal cues may help improve virtual communication. While both the sender and receiver are affected by the aforementioned factors, the primary burden appears to lie with the receiver, who faces reduced access to essential communicative cues [47,48,49,50].

These issues also seem to be reflected in physiological differences between in-person and online communication. Such physiological studies comparing in-person and virtual communication, however, remain limited. One reason for this limitation may be that the technologies available to detect biological signals, not least brain signals, are subject to motion artifacts, the unwanted signals caused by movement and muscle activity induced during natural conversation, from change in facial expression to head nodding, or body repositioning. These artifacts distort measurements, making it difficult to isolate clean physiological signals particularly in naturalistic experimental settings. Nonetheless, existing evidence suggests that virtual interactions are associated with reduced brain synchrony between participants [23,38] and diminished physiological responses, such as less pupil dilation when viewing faces [28]. In studies that use listening to mitigate head/muscular movement, where in-person and video lectures are compared, research has shown an increase in frontal theta activity during virtual meetings, indicating fatigue, and decreased levels of alpha activity in the parietal and occipital region, indicating lower levels of engagement. In addition, there is a decrease in heart rate variability (HRV), indicating stress or fatigue [30]. Higher HRV has been linked to better subjective conversation quality in face-to-face settings [51]. In contrast, a separate study that used text-based communication to avoid movement-related artifacts found no statistically significant differences in HR or GSR between in-person and virtual conditions, although both measures showed a trend toward higher values in the face-to-face setting [29]. Similarly, GSR responses to eye contact were found to be reduced during virtual meetings [27].

Overall, in-person communication tends to evoke stronger physiological and behavioral responses and greater interpersonal synchrony. In contrast, virtual settings tend to reduce these effects, likely due to limited access to spatial and nonverbal cues, leading to lower emotional engagement, arousal, connection, and trust.

## 3. Materials and Methods

This study was approved by the University of Southampton ethics committee in December 2023 (ERGO Application ID: 89571), and all procedures complied with the Declaration of Helsinki. Staff and students from the University of Southampton were invited to take part, and all individuals provided written informed consent prior to participation. Data collection occurred between 1.4.2024 and 1.7.2024. Sessions required approximately 90 minutes, including setup. A total of eighteen participants took part (14 male, mean age 34.8, SD 9.9), each receiving 20 GBP as compensation.

### 3.1. Experimental Task

The experimental protocol was an adapted version of a study by McFarland et al. [34], where respiratory markers during conversation were investigated. All stimuli (Texts, Videos) were selected so that they did not illicit any strong emotion to avoid contamination of unintended biomedical signals. For our version, we defined the following tasks:

- Baselines, each 2 min: These trials were used to familiarize the participant with the new laboratory environment and the experimental task. It was also used to record signals without interaction with another person.
  – Passive Baseline: The participant sat still and focused on the fixation cross on the screen.
  – Watch and Listen Baseline: The participant watched a video. We used the first 2 min from Huberman Lab Clip’s youtube channel with the title “How to Properly Hydrate & How Much Water to Drink Each Day|Dr. Andrew Huberman”. We picked this video because the creator talks directly to the listener, imitating a one-sided conversation.
  – Read Baseline: The participant read a text aloud after familiarizing themselves with it. We used the text “Kombat Kate” from the Cambridge English Assessment Example.
  – Free Talk Baseline: The participant talked about a topic of their choice without any restrictions.
- Dialogue, 5 min each: The dialogues took place between an experimenter and the participant, either in-person or virtually. The order of these two conditions alternated for each participant to avoid any temporal effects and always started with the reading task followed by free talk.
  – In-person Dialogue Reading: For this condition, we used ChatGPT (Version 3.5) to generate a simple dialogue between two people. While the text had sections of varying lengths for each speaker, the overall length was approximately the same for both, aiming to simulate a realistic conversation with balanced talking and listening parts. For this condition, the participant read the dialogue off their smart phone while trying to maintain eye contact with the experimenter when listening.
  – In-person Dialogue Free Talk: For this condition, participants were asked to choose a topic they are familiar with, one that is common enough for the experimenter to ask questions and share their own opinions. The goal for the conversation was again to have approximately equal speaking time for both the participant and the experimenter.
  – Virtual Dialogue Reading: For this condition, we used the same text from above but switched the roles in the dialogue. For this task, the experimenter left the room, and the talk was performed online using Zoom. For this condition, the participant read the dialogue off their smart phone as above.
  – Virtual Dialogue Free Talk: Same as In-person Dialogue Free Talk, but with a different topic and using Zoom.
- Anxiety, 5 min: This task was based on the InterneT based Stress Test for Social Anxiety Disorder (ITSSAD) by Huneke et al. [52]. Participants were instructed that they had five minutes to prepare for an online social interaction in which they would introduce themselves to a group of researchers. Prior work shows that this anticipation alone increases anxiety, so the full interview was not conducted in this pilot. After the five minutes, participants were debriefed and told that no interaction would take place. The experiment then concluded.

**Figure 1:**
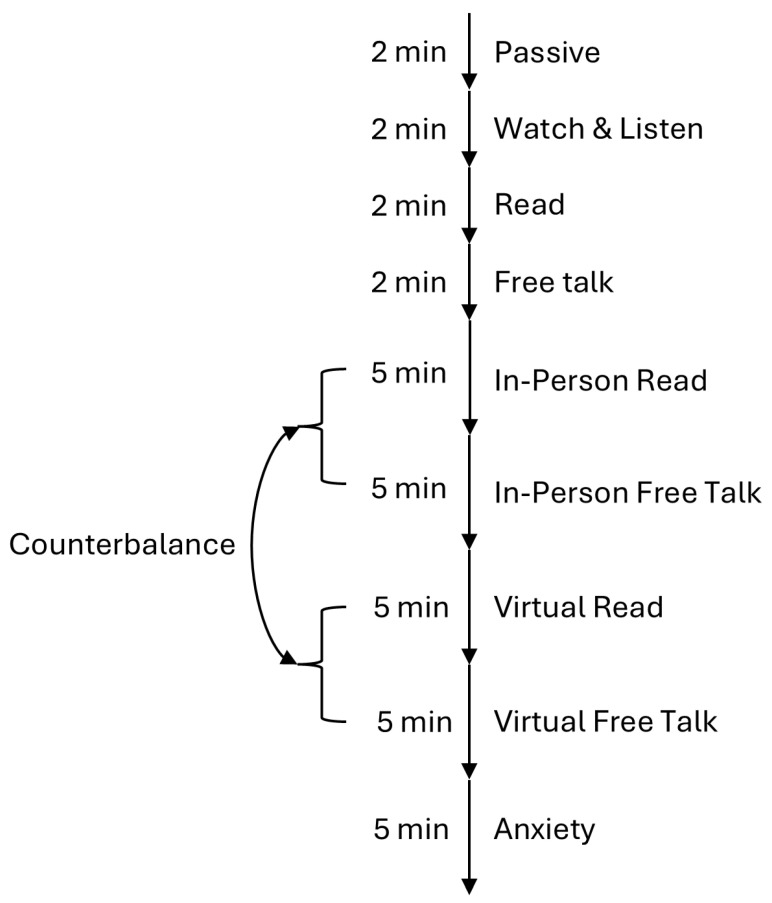
Flowchart of the experimental task.

The whole task is also visualized in Figure 1.

### 3.2. Experimental Procedure

Participants were sat comfortably while connected to a 27-electrode EEG, a 2-electrode electrocardiogram (ECG), a 2-electrode galvanic skin response (GSR), and a 4-electrode electrooculogram (EOG) using BrainProducts’ (Germany) “actiChamp plus” system. We used a MindMedia’s (Herten, Netherlands) “Nexus” respiration sensor with a custom connector to record respiratory rate; Pupil Lab’s (Gilching, Germany) “Neon” to track eye movements, pupil size, head acceleration, angular velocity, and orientation; Blue Microphones’(Westlake Village, CA, USA) “Blue Yeti” to record audio; and an ELP (Shenzhen, China) webcam model USBFHD01M-SFV to record video. EEG, ECG, EOG, GSR, and respiratory rate were sampled at 500 Hz; eye tracking and pupil size at 200 Hz; head acceleration, angular velocity, and orientation at 110 Hz; audio at 48 kHz; and video at 30 Hz. All signals were synchronized using LabStreamingLayer [53]. We do not describe video and audio further in the present paper since this analysis focuses on biosignals.

As part of the “ActiChamp” system, “ActiCap” slim wet electrodes were placed at positions FP1, F7, Fz, F3, FC5, T7, C3, Cz, TP9, CP1, P7, CP5, P3, O1, FP2, F8, F4, FC6, T8, C4, CP2, TP10, P8, CP6, P4, Pz, and O2, according to the International 10-20 system [54]. Two EOG electrodes were placed above (FP2) and below the right eye to capture the vertical eye movement signal, as well as two near the canthus of each eye for the horizontal signal. Two electrodes were placed at the insides of the elbow to get the ECG signal. Two GSR electrodes were placed on the index and middle finger of the participants’ non-dominant hand to obtain the GSR signal.

#### 3.2.1. Data-Recording Adjustments

Data analyses were adjusted based on signal quality and participant behavior as noted below.

- Exclusion of GSR: GSR data were discarded due to participant interference. Some individuals unintentionally interacted with the sensors during dialogue conditions, contaminating the data.
- Exclusion of EEG: EEG data were excluded due to motion artifacts caused by head movements, facial expressions, and muscle activity, which compromised signal integrity. However, minor power variations in the motor cortex suggested potential hand movements. To address this, video recordings were reviewed, and hand movements were documented. The EEG analysis is included in the Appendix A.
- Exclusion of Head Orientation: Head orientation was not analyzed because participant positioning differed between the virtual and in-person conditions.
- Inclusion of Hand Movements: Observed brain activity suggested the need to track hand movements, as demonstrated in the Appendix A. Only complete hand or arm movements were considered to avoid confounds from sensor interference. Hand movements were manually annotated from video recordings at a 1 Hz resolution.
- Exclusion of Audio and Video: Audio and video data were not analyzed, as the focus remained on biosignal and behavioral processing.

#### 3.2.2. Pre-Processing

A large dataset of physiological signals was recorded. Therefore, preprocessing and feature extraction focused on measures that either demonstrated statistical differences or were previously associated with human interaction in the literature. Given the study’s emphasis on interaction, the analysis was limited to the dialogue conditions “Reading” and “Free Talk”, and baselines were excluded. Results from the anxiety condition are presented in a different publication [55]. The following section describes the pre-processing steps for each of the selected signals. All filtering procedures utilized zero-phase filters to preserve temporal relationships. For signals without a defined frequency range, we applied a 50 Hz low-pass filter to avoid aliasing.

- ECG: The ECG signal was derived by subtracting both ECG electrodes and applying a band-pass filter (2–30 Hz), which captures the frequency range where most clinically relevant information in the QRS complex is found [56]. This was used to compute HR and HRV.
- Pupil Size: The pupil size signal was low-pass filtered at 50 Hz.
- Head Movements: Data from the 9-degree-of-freedom (DOF) inertial measurement unit (IMU), angular velocity, and acceleration in three axes each, were low-pass filtered at 50 Hz.
- Eye Tracking: Gaze position signals were low-pass-filtered at 50 Hz.
- Hand Movements: The manually recorded hand movements were used without any pre-processing.
- Respiratory Signal: A band-pass filter (0.1–0.5 Hz) was applied to the respiratory signal. The average adult breathing rate ranges from 12 to 20 breaths per minute (0.2–0.33 Hz) [57]. To avoid filter edge effects near the filter boundaries and to account for potentially elevated breathing rates in the anxiety condition, we used a range corresponding to 6 to 30 breaths (0.1–0.5 Hz) per minute.

Accurate alignment across electrophysiological, hand motion, and gyroscope recordings was necessary because these signals were captured on different devices. We used LSL timestamps instead of device specific timestamps and resampled all data streams to 100 Hz. The synchronized recordings were then segmented by task into 30 s windows with a 5 s step. This resulted in 55 windows for each dialogue condition.

#### 3.2.3. Feature Extraction

- HR: HR was extracted using a peak detection algorithm applied to the normalized negative derivative of the ECG signal of each window.
- HRV: HRV was extracted by calculating the standard deviation of all HR peak-to-peak intervals and dividing it by the root mean square of the peak-to-peak intervals.
- Pupil Size: The trace of the pupil size is corrected that the first window starts at zero. This was done to ensure that the distance between participant and experimenter for each condition was not influencing the outcome of the analysis. Each window was then featured by its mean.
- Respiratory Rate: A peak detection algorithm was used to extract the respiratory rate from the normalized respiratory signal.
- Head Movements: The power spectral density (PSD) of each signal was computed using Welch’s method with 1 Hz frequency bins.
- Gaze Position: The Gaze Position was featured by its variance.
- Hand Movements: The hand movements were featured by its percentage of time the hands were in motion.

The reading condition required participants to alternate between reading from their phones and maintaining eye contact with their conversation partners. This task-driven behavior introduces movement patterns that do not reflect natural social interaction. As a result, all movement-related measures (hand, eye, and head movements) and pupil size were excluded from analysis in this condition. The chosen features are based on physiologically plausible measures that are well established in the literature. Because this study relied on naturalistic interaction rather than a repeated block design, trial averaging could not be used to reduce noise. We therefore selected features that provide stable behavior at the single-window level and have clear physiological interpretations.

## 4. Results

Below, we report results for physiological and behavioral measures recorded during the two conversational tasks, “read aloud” and “free talk”, under both face-to-face and virtual conditions, as described in the Section 3.1. The recorded measures included HR, HRV, respiratory rate, pupil size, hand movements, horizontal and vertical eye gaze variance, and the PSD of head movements. While HR, HRV, and respiratory rate were analyzed across both tasks, movement-related metrics (hand, eye, and head movements) and pupil size were only assessed during the free talk condition, as the reading task involved alternating gaze between reading of their phone and watching the conversation partner. EEG and GSR data were excluded due to muscle-related artifacts and unintentional interaction with the GSR sensor. However, minor power variations over the motor cortex indicated potential hand movement, which was further examined using video recordings. A detailed discussion of the EEG analysis and exclusion rationale is provided in the Appendix A.

Each biosignal was examined independently. The figures in the Section 4 use a uniform layout. The left panel depicts the physiological measure over time from 30 to 300 s. Yellow regions mark intervals in which the two groups differed significantly. Because EEG [58], ECG [59], and eye gaze dynamics [60] are non-stationary and non-linear, we evaluated group differences with a non-parametric Wilcoxon signed-rank test. Given the small sample size of this pilot study, we did not apply corrections for multiple comparisons in the time series analysis. These outcomes are exploratory and indicate potential time periods worth investigating in larger follow-up studies. The right panel of each figure shows a boxplot summarizing the average value of the measure across the entire window, together with the p-value for the group comparison based on the same Wilcoxon signed-rank test. For the PSD analysis, significant differences in frequency bands were observed for head movements, and these p values were corrected using the Benjamini–Hochberg procedure.

**Figure 2:**
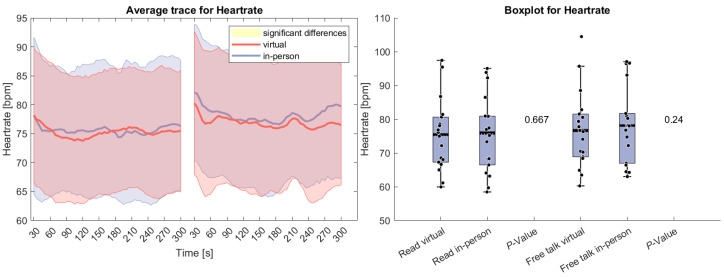
(**Left side**): Average trace and standard deviation of heart rate during the reading and free-talk tasks. The in-person trace is shown in blue, while the virtual trace is shown in red. The blue and red shaded areas represent the standard deviation for in-person and virtual sessions, respectively. Yellow shading, if visible, indicates significant differences between the two conditions. (**Right side**): Box plot for heart rate averaged over each task for virtual and in-person condition with a *p*-value for a Wilcoxon signed-rank test.

Figure 2 presents HR data for the reading and free-talk tasks in both the virtual and in-person conditions. On average, HR was higher in the in-person condition compared to the virtual condition for both tasks. The difference was more pronounced during the free-talk task but did not reach statistical significance in either case. At the start of both tasks, HR initially declined, a common effect observed in physiological studies. This decrease is likely due to the orienting response [61], which occurs when individuals react to a change in their environment with an initial state of heightened arousal before adaptation.

**Figure 3:**
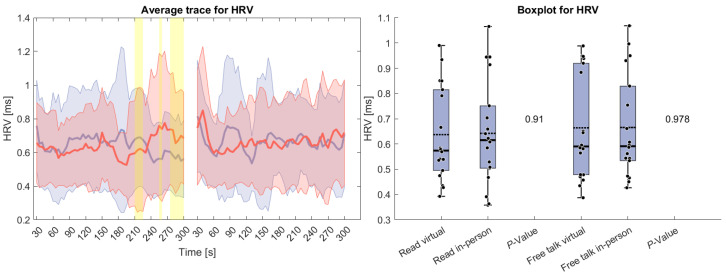
(**Left side**): Average trace and standard deviation of heart rate variability during the reading and free-talk tasks. The in-person trace is shown in blue, while the virtual trace is shown in red. The blue and red shaded areas represent the standard deviation for in-person and virtual sessions, respectively. Yellow shading, if visible, indicates significant differences between the two conditions. (**Right side**): Box plot for heart rate variability averaged over each task for virtual and in-person condition with a *p*-value for a Wilcoxon signed-rank test.

Figure 3 shows HRV, which remained relatively stable throughout both tasks. Only at the end of the reading task can a difference be seen, where the HRV for the virtual condition increases and for the in-person decreases. However, this variation did not meaningfully impact the overall measure, and no statistically significant differences were found.

**Figure 4:**
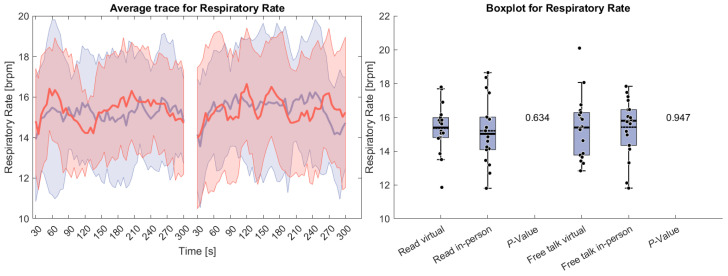
(**Left side**): Average trace and standard deviation of respiratory rate during the reading and free-talk tasks. The in-person trace is shown in blue, while the virtual trace is shown in red. The blue and red shaded areas represent the standard deviation for in-person and virtual sessions, respectively. Yellow shading, if visible, indicates significant differences between the two conditions. (**Right side**): Box plot for the respiratory rate averaged over each task for virtual and in-person condition with a *p*-value for a Wilcoxon signed-rank test.

Figure 4 depicts respiratory rate during both tasks. No significant differences were observed between the conditions. Unlike HR, the respiratory rate initially increased, likely due to a transient elevation in the breathing rate as participants began speaking. This suggests that participants did not start conversing immediately but required a brief adjustment period before engaging in speech.

**Figure 5:**
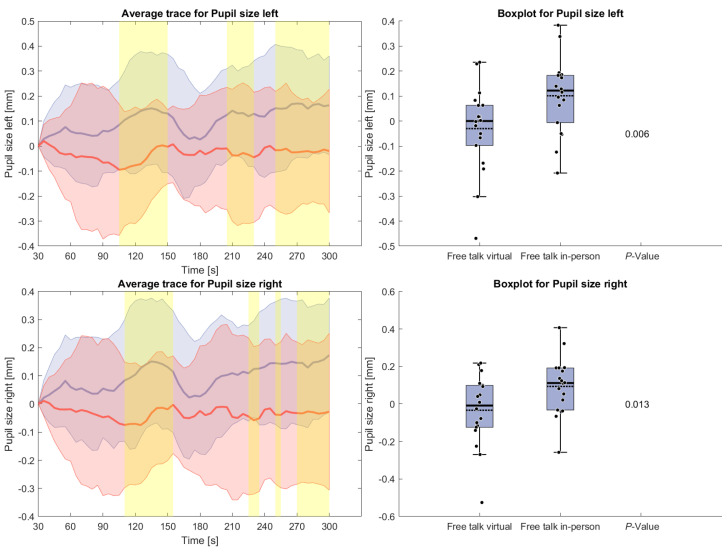
(**Left side**): Average trace and standard deviation mean pupil size during the reading and free-talk tasks. The in-person trace is shown in blue, while the virtual trace is shown in red. The blue and red shaded areas represent the standard deviation for in-person and virtual sessions, respectively. Yellow shading, if visible, indicates significant differences between the two conditions. (**Right side**): Box plot for pupil size averaged over the free-talk task for virtual and in-person condition with a *p*-value for a Wilcoxon signed-rank test.

The following measures will only present results for the free-talk condition because of the interference of the reading task with the signal acquisition as mentioned above. Figure 5 shows pupil size during the free-talk task. After an initial rise from the zero baseline, pupil size remained larger in the in-person condition compared to the virtual condition. A brief drop for the in-person average occurred around 180 s, but overall, the increase was statistically significant.

**Figure 6:**
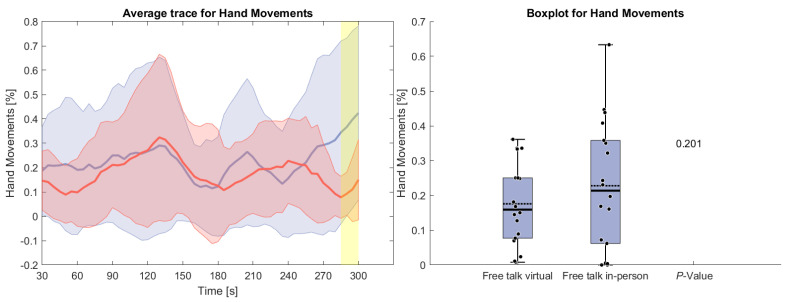
(**Left side**): Average trace and standard deviation of counted hand and arm movements during the reading and free-talk tasks. The in-person trace is shown in blue, while the virtual trace is shown in red. The blue and red shaded areas represent the standard deviation for in-person and virtual sessions, respectively. Yellow shading, if visible, indicates significant differences between the two conditions. (**Right side**): Box plot for counted hand and arm movements averaged over each task for virtual and in-person condition with a *p*-value for a Wilcoxon signed-rank test.

Figure 6 displays the counted hand movements of participants during the free-talk task based upon the detected brain activity. It can be seen that the hand movement during the in-person condition hand movements were happening more often, especially at the beginning and the end of the task. However, these results do not show any statistical significance.

**Figure 7:**
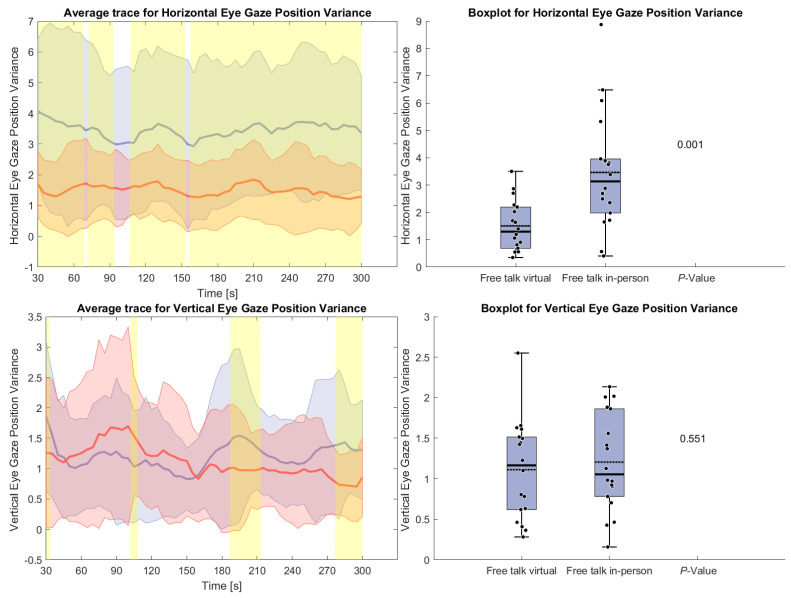
(**Left side**): Average trace and standard deviation of horizontal and vertical eye gaze variance during the reading and free-talk tasks. The in-person trace is shown in blue, while the virtual trace is shown in red. The blue and red shaded areas represent the standard deviation for in-person and virtual sessions, respectively. Yellow shading, if visible, indicates significant differences between the two conditions. (**Right side**): Box plot for the horizontal and vertical eye gaze variance averaged over the free-talk task for virtual and in-person condition with a *p*-value for a Wilcoxon signed-rank test.

Figure 7 shows the variance of horizontal and vertical eye gaze positions during the free-talk task. The horizontal gaze variance was significantly higher in the in-person condition throughout the task, indicating greater eye movement variability. In contrast, vertical gaze variance remained similar across conditions, with no significant differences.

**Figure 8:**
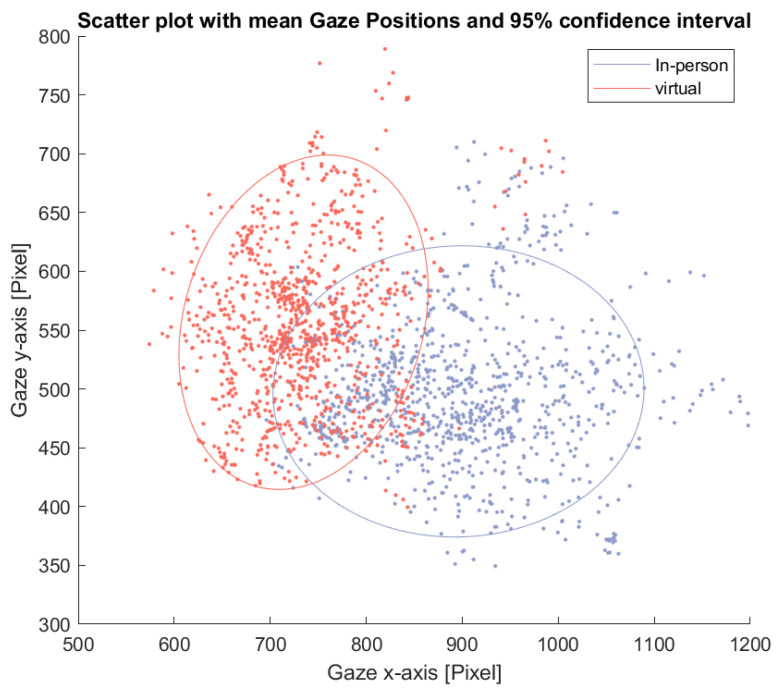
Scatter plot of the average eye gaze position across participants for each time window during the free-talk task. The ellipses indicate the 95% confidence interval for each condition.

This pattern is further illustrated in Figure 8, which shows a scatter plot of average eye gaze positions for each window and participant. The ellipses represent the 95% confidence interval for each condition. The in-person condition shows a wider spread in average gaze position along the horizontal axis.

**Figure 9:**
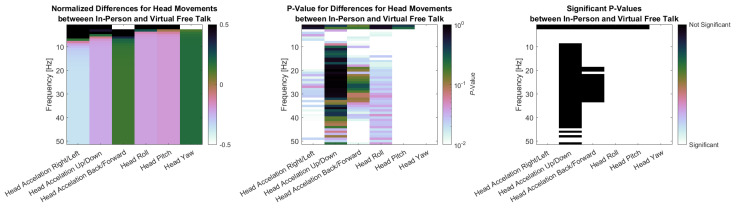
(**Left side**): Normalized differences between head movement PSD signals in-person and virtual for the free-talk task. (**Middle**): *p*-values for Differences between head movement PSD signals in-person and virtual for the free-talk task. (**Right**): Significance for head movement PSD signals comparing in-person and virtual for the free-talk task.

Finally, Figure 9 presents the PSD results for head movements in three subplots. The first plot shows the normalized difference in PSD between the in-person and virtual conditions during the free-talk task. Head acceleration in the right/left and up/down directions, look similar as they show larger power for the in-person condition for low-frequency areas (up to 7 Hz and 5 Hz respectively) and then a lower power for higher frequencies. Head Acceleration Back/Forward shows the reverse with lower Power for 0–2 Hz and higher Power for higher Frequencies. The angular velocities show a similar pattern. Roll and Pitch show higher power for the in-person condition up to 2 Hz and lower power above, while Yaw show lower the exact opposite. The second plot displays the p-values for these differences, calculated using the Wilcoxon signed-rank test with Benjamini–Hochberg correction. The third plot highlights the significant p-values for easier identification. Nearly all signals showed significant differences, except for very low-frequency components of back/forward and Yaw (0 Hz), head acceleration in the up/down direction above 10 Hz, and head acceleration in the back/forward direction between 20 and 30 Hz.

Head acceleration in the right/left and up/down directions showed higher power in the in-person condition at lower frequencies (up to 7 Hz and 5 Hz, respectively), but lower power at higher frequencies. In contrast, back/forward acceleration displays the opposite pattern, with reduced power below 2 Hz and increased power at higher frequencies. A similar trend was observed in angular velocities. Roll and pitch showed greater power during in-person interaction up to 2 Hz, while yaw displays the reverse. The second plot presents the *p*-values from Wilcoxon signed-rank tests with Benjamini–Hochberg correction, and the third highlights statistically significant differences. Most frequency components revealed significant differences, with exceptions primarily at very low frequencies at 0 Hz, as well as high-frequency components above 10 Hz (up/down) and 20–30 Hz (back/forward).

## 5. Discussion

This study investigated how in-person and virtual communication differ in naturalistic settings. Its main contributions were the integration of behavioral and physiological measures in a multimodal setup, the examination of unscripted interaction that reflects everyday communication, and the inclusion of both sender and receiver perspectives to provide a more complete view of the interaction process.

We found significantly larger pupil dilation and greater horizontal eye movement variance during in-person interactions. Head movements also differed significantly in PSDs for acceleration and angular velocity across all three axes. Head acceleration in right/left and up/down, as well as angular movement in roll and pitch, showed higher power in low-frequency components and lower power in high-frequency components for the face-to-face condition. Most of these components showed significant differences, except up/down acceleration at frequencies above 10 Hz and very low frequencies (0–1 Hz). Meanwhile, head acceleration in back/forward and angular velocity in yaw showed the opposite with lower non-significant power for very low frequencies (0–1 Hz) and higher power for frequencies above that. Although heart rate and hand movement levels were higher in-person, these differences were not statistically significant, and no effects were observed for heart rate variability or respiratory rate. GSR was excluded because participants interacted subconsciously with the sensor during the study, leading to unusable recordings. EEG spectral power was also removed from the main analysis. Although we observed apparent differences in the beta and gamma ranges during the free-talk task, activity above 20 Hz is typically dominated by EMG contamination. After applying a denoising algorithm, the remaining signal showed only minor activity over motor areas, consistent with hand movements, and these effects were not significant. The complete analysis is shown in the Appendix A.

The physical constraints of virtual communication, such as reduced mobility and limited gesture visibility, likely contributed to the significantly lower eye movement levels observed [23,45], and may also explain the non-significant reduction in hand movements. The lack of significant vertical movement differences above 10 Hz is expected, as participants remained seated, allowing for only a limited variation in body height. Participants in virtual settings also likely limited all larger movement to stay within the camera frame, which may have led them to compensate with smaller movements that produced more higher frequency changes.

In our study, we found increased pupil dilation for the in-person condition, which aligns with prior research showing that arousal is higher during face-to-face interactions [29], and that increased arousal is associated with greater pupil size [62]. This also agrees with a study that showed increased pupil dilation when viewing faces in-person versus on screen [28]. That same study reported broader brain activation and larger event-related neural responses in the in-person condition, consistent with trends observed in our EEG data analysis in the Appendix A. However, due to our minimally restrictive, naturalistic setup, we cannot confidently attribute the observed effects solely to neural activity. Movements and facial expressions during conversation introduce electromyographic (EMG) artifacts: high-amplitude broadband signals that overlap with EEG frequencies and are difficult to fully remove without distorting the underlying brain signals [63]. Given that participants moved more during face-to-face interactions in our study and previous research has shown increased coordination in such settings, the resulting muscle-related artifacts, especially when movements are synchronized, could partly explain the higher EEG synchrony observed in previous studies on in-person communication [23,38]. We also observed a non-significant increase in heart rate in the in-person condition, which is consistent with previous findings [29], and might reflect both greater physical activity and heightened engagement and arousal typically associated with face-to-face interactions.

In contrast to previous findings, we did not observe significant HRV differences between in-person and virtual communication, possibly due to the short duration of our five-minute trials, compared to longer exposures in other studies (e.g., 50-min lectures or multi-day recordings) [30,51]. Nevertheless, this result is consistent with our respiratory data, which also showed no differences between conditions, aligning with the known relationship between HRV and respiratory rate [64].

Our findings suggest that both sender and receiver constraints impact virtual communication. While virtual communication technologies are relatively new, limitations such as restricted sender movement and reduced receiver access to nonverbal cues have been recognized since early telemedicine research [65]. Various strategies exist to mitigate these effects, such as removing or hiding the camera feedback to prevent distractions for the sender [44] or improving field of view to enhance gesture visibility for the receiver [66].

This study has several limitations. Because it was designed as a pilot study, we focused on time- and frequency-domain features that are established in literature and offer clear physiological interpretability. The naturalistic interaction design prevented the use of repeated block structures, limiting our ability to average out noise and making more advanced analyses less suitable at this stage. Some modalities, such as GSR and high-frequency EEG activity, had to be excluded due to motion-related artifacts. Future work will address these limitations by using larger samples, incorporating richer feature domains, and applying improved denoising methods or task designs that reduce motion-induced noise.

## 6. Conclusions

In summary, our results show mostly significant differences in the behavioral measures between in-person and virtual communication, indicating clear modality-dependent changes in how people interact. These differences cannot be primarily attributed to receiver-side limitations, such as reduced access to non-verbal cues due to restricted visual fields, limited gesture visibility, or disrupted eye contact caused by camera positioning. Instead, our findings also point to sender-side constraints: participants in virtual settings showed markedly reduced horizontal eye movement variance, head and body motion, particularly in lateral acceleration and angular momentum, and a non-significant decrease in hand gestures. These patterns likely arise from the physical restrictions imposed by the virtual setup, such as the need to stay centered in the camera frame. Overall, virtual communication appears to constrain both the expression and perception of non-verbal behavior, potentially leading to a diminished sense of social connection.

Our findings emphasize that both sender and receiver constraints impact virtual communication, and addressing both is crucial for improving system interaction design. Addressing the established constraints of virtual communication and revisiting and adapting established mitigation approaches in light of current findings could help improve the quality and naturalness of virtual interactions.

## Figures and Tables

**Figure A1:**
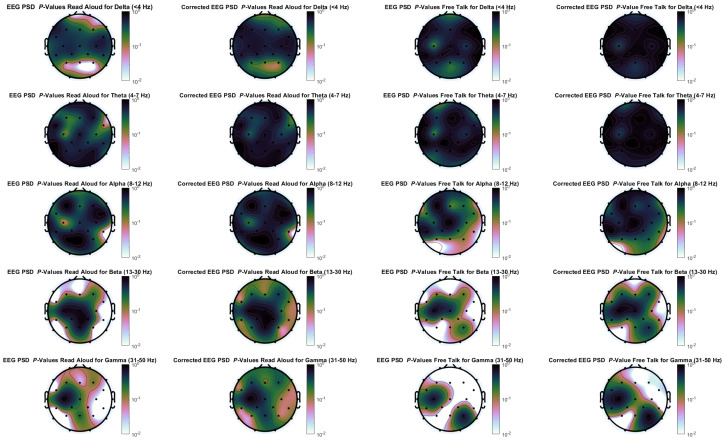
EEG power spectral density *p*-values for differences between in-person and virtual condition for the frequency bands delta, theta, alpha, beta and gamma for both tasks. The left side for each task is without false positive correction, while the right side uses the Benjamini–Hochberg correction.

**Figure A2:**
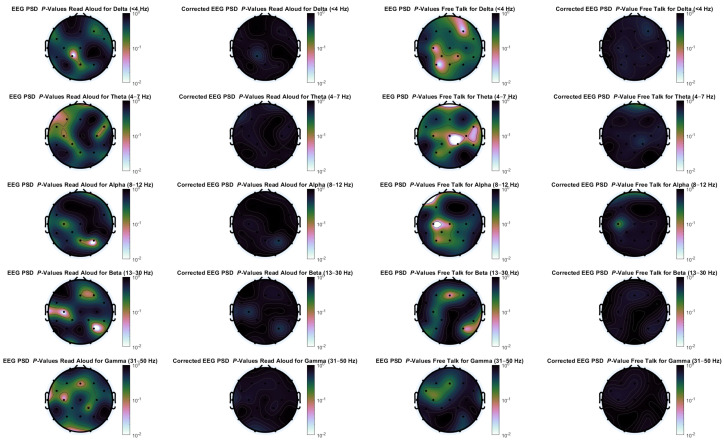
Denoised EEG power spectral density *p*-values for differences between in-person and virtual condition for the frequency bands delta, theta, alpha, beta, and gamma for both tasks. The left side for each task is without false positive correction, while the right side uses a Benjamini-Hochberg correction.

## Data Availability

The original data presented in the study are openly available at https://eprints.soton.ac.uk/503817/, (accessed on 31 October 2025).

## Abbreviations

The following abbreviations are used in this manuscript:

HR	Heart Rate
GSR	Galvanic Skin Response
EEG	Electroencephalography
fNIRS	Functional Near-Infrared Spectroscopy
DOF	degree of freedom
IMU	Inertial Measurement Unit
HRV	Heart Rate Variablity
ITSSAD	InterneT-based Stress Test for Social Anxiety Disorder
ECG	Electrocardiogram
EOG	Electrooculogram
PSD	Power Spectral Density
EMG	Electromyography
STFT	Short-Time Fourier Transform

## Appendix A

This section outlines the preprocessing, feature extraction, and analysis steps for EEG data and explains why the observed results are likely driven by movement and muscle artifacts. Additionally, we justify the inclusion of hand movements as an additional measure.

EEG signals were recorded from 27 channels, referenced to the average of electrodes TP9 and TP10, resulting in a total of 25 channels. EOG signals were used for artifact removal. Vertical and horizontal EOG channels were derived by subtracting pairs of vertical and horizontal EOG electrodes, respectively. EEG data were filtered between 0.5 and 50 Hz, with an additional 50 Hz notch filter applied to remove power-line interference. Eye movement artifacts were mitigated using a linear regression model, where vertical and horizontal EOG signals were predicted from each EEG channel and subtracted [67]. This step was necessary to isolate brain activity from ocular artifacts. A common average reference filter [68] was then applied before computing the PSD using Welch’s method with 1 Hz frequency bins.

Figure A1 presents *p*-values for differences in EEG activity between the in-person and virtual conditions across both tasks, computed using a Wilcoxon signed-rank test with and without Benjamini-Hochberg correction. Using standard EEG processing as described above, significant differences were primarily observed in the beta and gamma frequency ranges, with some uncorrected significance in the delta range for the read aloud condition. Notably, after correction, the free-talk task still showed significant differences, whereas the read aloud task no longer exhibited meaningful significance. However, these results should be interpreted with caution. Above 20 Hz, EMG activity tends to overwrite EEG recordings, making it likely that these differences reflect muscle rather than brain activity [69]. This interpretation aligns with our head and eye movement analysis (Figure 8 and Figure 9), which shows greater involuntary movement during the free-talk task. Therefore, the observed EEG differences are more likely driven by muscle activity than by meaningful neural responses.

To assess the influence of muscle activity during the free talk and reading task, a denoising procedure analogous to the one used for EOG artifact correction was applied. Since the EEG analysis focused on PSD features, the time-domain EEG signals were first transformed into the frequency domain using a Short-Time Fourier Transform (STFT). Phase information was preserved, while the power spectrum was adjusted using a method similar to the time-domain EOG correction. Movement-related noise was predicted using data from the 9-DOF IMU—specifically, acceleration and angular velocity along three axes. These signals were segmented using the same window length and overlap as the EEG data. From each segment, the mean, variance, and PSD in 1 Hz frequency bins were computed. A linear regression model was then constructed to predict EEG power at each frequency band using all extracted movement features—mean, variance, and full-spectrum PSD—from the IMU signals. The predicted components were subtracted from the EEG power spectra to suppress contributions from muscle and movement artifacts. All movement signal frequencies were used to predict each EEG frequency, rather than matching frequencies directly. This approach was chosen to account for non-linear and cross-frequency relationships between movement and EEG activity, particularly in the higher frequency range where EMG contamination is most pronounced. Finally, the denoised power spectra, along with the preserved phase information, were used to reconstruct the cleaned EEG signal via inverse STFT.

The results of the denoised PSDs are presented in Figure A2. After applying correction for multiple comparisons, no significant differences were observed across any channel or frequency band, highlighting the substantial influence of movement-related artifacts on the original EEG signals. Before correction, several channel-frequency combinations showed significant p-values; however, it remains unclear whether these reflect genuine neural activity or residual artifacts not fully eliminated by the denoising procedure. The only neural signal that could be reliably verified was increased alpha-band activity at electrode C3 for the free-talk task. This corresponds to the left sensorimotor cortex and aligns with mu rhythm (9–11 Hz) activity, which is typically associated with motor processes such as right-hand movement. Given that the majority of participants were right-handed, hand movements were included as an additional behavioral measure in this study.
